# The Effects of Exercise on Inhibitory Function Interventions for Patients With Major Depressive Disorder (MDD): A Systematic Review and Meta‐Analysis

**DOI:** 10.1002/brb3.70178

**Published:** 2024-12-31

**Authors:** Zhihui Xu, Cong Liu, Peng Wang, Xing Wang, Yuzhang Li

**Affiliations:** ^1^ School of Physical Education Shanghai University of Sport Shanghai China

**Keywords:** exercise, depression, inhibitory functions, systematic review

## Abstract

**Background:**

Systematic Review of The effects of exercise on inhibitory function interventions for patients with major depressive disorder.

**Methods:**

We searched PubMed, Web of Science, EMbase, The Cochrane Library, China National Knowledge Infrastructure (CNKI), Wanfang Database, and China Science and Technology Journal Database (CQVIP) for randomized controlled trials (RCTs) investigating the impact of exercise on inhibitory function in MDD patients, from database inception to July 2024. Two researchers independently evaluated the quality of the included studies using the Risk of Bias (ROB 2.0) tool. Evidence quality was assessed with the GRADE profiler software, and effect sizes were combined using Stata 17.0 software to create forest plots, test for publication bias, and perform sensitivity analyses.

**Results:**

A total of nine RCTs involving 1038 participants from six countries, published between 2001 and 2022, were included. The average age of participants in both the experimental and control groups was 45 years. Meta‐analysis results indicated that exercise significantly improves inhibitory function in MDD patients, with a combined effect size (SMD = 0.48, 95% CI = 0.18–0.77, *p* < 0.001). Subgroup analysis showed that exercise had a statistically significant effect on inhibitory control in MDD patients, with an effect size (SMD = 0.563, *p* < 0.001). Regarding exercise elements, other types of exercise (resistance exercise RE, mixed exercise ME), duration greater than 45 min, intervention period of ≤12 weeks, frequency of two times per week, and low intensity were found to be more effective, all with statistical significance (SMD = 0.863, *p* < 0.001; SMD = 0.936, *p* < 0.001; SMD = 0.525, *p* = 0.002; SMD = 0.682, *p* = 0.004; SMD = 0.94, *p* = 0.00).

**Conclusion:**

Based on the International Classification of Diseases (ICD) and the Diagnostic and Statistical Manual of Mental Disorders (DSM) classification systems, a research framework for exercise interventions on executive function in MDD patients was constructed, demonstrating that exercise can improve inhibitory function in MDD with high evidence quality. Our study found that other types of exercise (RE or ME), intervention duration of >45 min, intervention period of ≤12 weeks, frequency of two times per week, and low intensity are more effective for improving inhibitory function in MDD patients. While the overall methodological quality of the literature was good, high heterogeneity existed among studies. Subgroup analysis suggested that sources of heterogeneity included measurement tools, exercise types, exercise intensity, duration, and frequency. Sensitivity analysis indicated that exercise duration and period might be causes of heterogeneity. This study has some limitations as the included literature did not consider disease duration, depression severity, or categorize age groups. However, the findings provide strong evidence for clinical practice and future research on the beneficial effects of exercise on inhibitory function in MDD patients.

**Trial Registration:**

CRD42023480371

## Graphical abstract

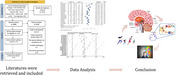

## 1 Introduction

Depression, also known as depressive disorder, is a mental disorder characterized by high morbidity, a high clinical cure rate but low treatment acceptance rate, and a high relapse rate (Zhang Shen‐Shuai et al. 2023). Typical symptoms include depressed mood, loss of interest or pleasure, and in severe cases, self‐harm or suicidal thoughts and behaviors (Wang et al. 2024). According to the World Health Organization, around 350 million people globally suffer from depression, leading to up to 1 million suicides each year. By 2030, depression is expected to become the leading cause of the global disease burden (Miret et al. 2013; Tian et al. 2013). As the prevalence of depression increases, it will impose a significant societal burden in the future (Peng et al. 2015).

Impaired inhibitory functioning is a key feature of depression (Semkovska et al. 2019). Rock et al. (2013) reported that approximately two‐thirds of depressed individuals exhibit deficits in inhibitory functioning. Inhibitory functioning, as one of the important indicators of the development of higher order cognitive functioning (Sun Wenjing and Di 2012), plays an important role between cognitive bias and cognitive impairment in depressive symptomatic groups, and deficits in inhibitory functioning have become a common clinical manifestation in most patients with mental disorders. Inhibitory functioning primarily refers to the blocking of access to information that may be partially activated that is not relevant to the target and the inhibition of stimuli that are not relevant to the task at hand (Depression Research Group 2020). Research (Amatriain‐Fernández, García‐Noblejas, and Budde 2021) suggests that individuals with depressive symptoms have significant deficits in inhibitory functioning, which may be related to cognitive and emotional symptoms in individuals with depressive symptoms. It has been shown that depressed individuals tend to perform poorly on the Stroop task, possibly due to difficulties in suppressing attention to negative words in this population (Gotlib and Joormann 2010). Additionally, depressed individuals also show difficulties with response inhibition in the Go/No‐Go task and the Stop Signal task, which may be related to their ability to regulate their emotions (Joormann 2010).

Exercise therapy has been one of the important non‐pharmacological interventions in antidepressant treatment in recent years, showing favorable antidepressant effects (Harvey et al. 2018) and an improved inhibitory functioning (Brush et al. 2022), but the specific effect of the impact has been inconsistent. A cross‐sectional study found a strong interrelationship between exercise participation, behavioral inhibition, and depressive symptoms, with exercise participation increasing behavioral activation systems and improving depressive symptoms (Fang et al. 2022). In longitudinal experiments (Hu et al. 2020; Miller et al. 2020), it was found that patients' physical fitness improved after exercise, which led to improvements in inhibitory functioning. Systematic evaluations and meta‐analytic studies have shown that exercise has the effect of significantly improving inhibitory functioning in depressed patients (Kvam et al. 2016). Even low‐intensity exercises like walking and yoga have been found to positively affect depression (Cramer et al. 2013). However, some studies argue that physical exercise does not significantly impact inhibitory functioning in adult depressed patients compared to controls (Contreras‐Osorio et al. 2022).

Based on this, combing through previous studies, we know that there is impaired inhibitory functioning in the depressed population and that exercise is an effective means of improving depression, but the total effect on inhibitory functioning in MDD is still controversial. Second, we found that previous studies did not have subgroup analyses based on elements such as exercise duration, exercise period, and exercise frequency, and the optimal intervention program needs to be further explored. Therefore, the present study will summarize and extract the evidence from previous randomized controlled trials and conduct a meta‐analysis of the inhibitory function of exercise in patients with depression to investigate whether there is a “dose effect” of the five elements of exercise on each outcome indicator. This will provide a more accurate exercise intervention program for clinical practice and future researchers to improve the inhibitory function of patients with depression.

## 2 Method

This study follows the PRISMA (Preferred Reporting Items for Systematic Reviews and Meta‐Analyses) guidelines to ensure research transparency (Page et al. 2021b). The study protocol has been registered on PROSPERO (https://www.crd.york.ac.uk/PROSPERO/) with registration number CRD42023480371.

### 2.1 Literature Search Strategy

The study followed the requirements of the Systematic Reviews 2020 (Page et al. 2021a) version and the guidelines of PRISMA 2020 (Radua 2021). Randomized controlled trials of exercise on inhibitory function in depressed patients were searched independently by two researchers using a computer to search seven major databases, China Knowledge, Wanfang, Wipro, PubMed, Embase, The Cochrane Libraryhe, and Web of Science, with the search date of each database established until July 2024. The search was conducted by combining subject terms with free words, using the Boolean symbols “AND” and “OR” for combinatorial concatenation, and was determined after repeated pre‐testing. EndNote 20 was used for initial screening, and then the full text was read for fine screening. In case of disagreement between the two researchers, the decision was discussed with a third researcher. This was supplemented by tracking down relevant systematic reviews and references to the included literature. The search procedure for each search library is shown in the Appendix. The Chinese search terms included “exercise/aerobic exercise/anaerobic exercise/resistance training/strength training/ball sports/physical and mental exercises/martial arts/yoga/depression/depression/inhibitory function/cognitive function/cognition/executive function/inhibitory/control,” and the English search terms included “Exercise/Aerobic exercise/physical exercise/resistance training/training/physical activity/sport/Anaerobic sports/Ball sports/Physical and Mental Exercise/Martial Arts/yoga/Depression/Depressive disorder/Depressive symptom/Emotional depression/Depressive neurosis/Endogenous depression/Unipolar depression/Inhibition function/Cognition/Cognitive performance/Cognitive/executive function/Randomized controlled trial/Randomized/controlled/Trial.”

### 2.2 Literature Eligibility Criteria

The inclusion criteria were based on the PICOS framework to systematically evaluate and analyze the effects of exercise on inhibitory function in depressed patients, aligning with the International Classification of Disease (ICD) and the Diagnostic and Statistical Manual of Mental Disorders (DSM) criteria. Both experimental and control groups consisted of depressed patients meeting these diagnostic criteria. Details of exercise modalities and outcome indicators are provided in the Appendix. Exclusion criteria included non‐depressed patients, inconsistent intervention content, non‐extractable data, and non‐research literature types such as reviews or conference papers.

### 2.3 Data Extraction

Extracted information included basic study details (author, year, nationality, age, and sample size), experimental characteristics (exercise type, intensity, duration, and frequency), and outcome indicators. Missing or unclear data were requested from original authors via email. Authors declared no conflicts of interest.

### 2.4 Quality Assessment

The quality of the literature was assessed using the latest revised version of the Cochrane risk of bias assessment tool (ROB 2.0) (Cumpston et al. 2019; Zhu Tao et al. 2022), with two researchers conducting the quality evaluation independently. For randomized controlled trials (RCTs), a score of “Low” in all domains indicated a low risk of bias. If at least one domain was rated “Some concerns” and none were rated “High risk,” it was classified as having a moderate risk of bias. If at least one domain was rated “High risk,” it was considered a high risk of bias. In case of disagreement, a third researcher made the final decision.

### 2.5 Evaluation of Quality of Evidence for Outcomes

The GRADE profiler software was used to evaluate the quality of evidence based on five downgrading factors: publication bias, inconsistency, imprecision, indirectness, and study limitations. Evidence was categorized into four grades: high, intermediate, low, and very low. Quality ratings were independently conducted by two researchers, with a third researcher resolving any disagreements.

### 2.6 Statistical Methods

Stata 17.0 software was used for effect size combination, publication bias, and subgroup analysis. Cohen's *d* was used for effect sizes, adjusting for measurement tool direction inconsistencies. Effect sizes were categorized as small (<0.2), small‐to‐medium (0.20–0.49), medium (0.50–0.79), and large (≥0.8) (Jacob 1992). Heterogeneity was measured using the *I* ^2^ statistic, with thresholds of 25%, 50%, and 75% indicating low, medium, and high heterogeneity, respectively (Higgins et al. 2003). A random effects model was used for high heterogeneity; otherwise, a fixed effects model was applied.

## 3 Results

### 3.1 Literature Search Results

**FIGURE 1:**
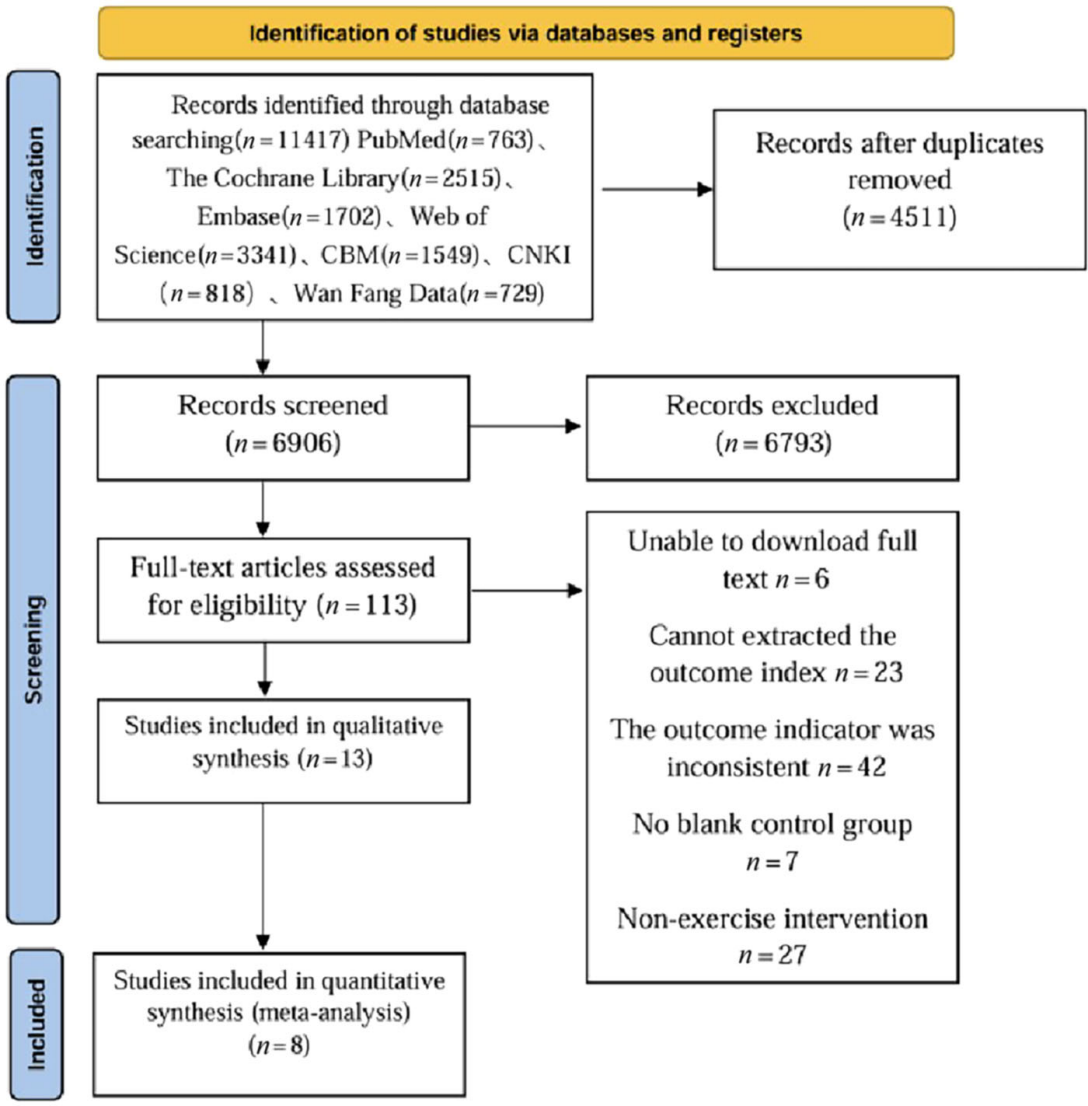
Literature retrieval process.

We identified 11,417 articles in PubMed, 2515 in The Cochrane Library, 1702 in Embase, 3341 in Web of Science, 1549 in CQVIP, 818 in CNKI, and 729 in Wanfang. After removing 4511 duplicates, 6793 articles were excluded based on title and abstract screening in EndNote. Following full‐text reading, six articles were inaccessible, 23 had non‐extractable data, 42 did not meet outcome criteria, seven lacked blank controls, and 27 were non‐exercise interventions. Eight articles were included in the meta‐analysis, as shown in Figure 1.

### 3.2 Characteristics of Included Literature

As shown in Tables 1 and 2, this meta‐analysis included eight studies (Bruchle et al. 2021; Hoffman et al. 2008; Imboden et al. 2020; Khatri et al. 2001; Krogh et al. 2012; Olson et al. 2017; Leleikiene et al. 2018; I. Zhang et al. 2022), encompassing nine RCTs. The studies were published between 2001 and 2022 and involved a total of 1038 depressed patients, with 563 in the experimental group and 475 in the control group. All subjects were diagnosed with depression, and the forms of exercise included aerobic exercise (AE), resistance training (RE), stretching (STM), and multiform exercise (ME). The intervention duration was mostly around 45 min, with the longest being 90 min (including time for warm‐up and relaxation exercises, with actual exercise duration around 30 min). The frequency of interventions was up to three times per week, with the longest intervention period being 16 weeks and the shortest 3 weeks. Exercise intensity was categorized according to the American College of Sports Medicine (Armstrong et al. 2006) based on maximum heart rate (HRmax) into high, medium, and low intensity. The outcome measures focused on inhibitory function, assessed using three tools: the Stroop test, the Go/No‐Go test, and the Flanker test.

**TABLE 1:** Table of basic characteristics included in the study (*n* = 9).

Study	Country	Sample E/C	Age (years)		Intervention characteristics
			E	C	Interference list
Hoffman et al. 2008	America	104/49	51.7 ± 7.6	51.7 ± 7.6	Stroop Color and Word Test—Trail Making Test (TMT)
Imboden et al. 2020	Switzerland	22/20	41.3 ± 9.2	38.3 ± 13.4	GO/No‐Go ‐TAP V 2.3 (Testbatterie zur Aufmerksamkeitsprüfung)
Khatri et al. 2001	America	42/42	56.73 ± 6.45	56.73 ± 6.45	Stroop Color and Word Test—Trail Making Test Part B.
Krogh et al. 2012	Denmark	56/59	39.7 ± 11.3	43.4 ±11.2	Stroop Attention—Digit Span Test
Leleikiene et al. 2018	Lithuania	13/13	54.1 ± 18.3	55.1 ± 16.5	GO/No‐Go Task—the reaction time and the correct response rate
Olson et al. 2017	America	15/15	18–30	18–30	flanker task of Eriksen—the reaction time and the correct response rate
I. Zhang et al. 2022	China	20/19	47.20 ± 6.99	54.16 ± 6.09	Chinese Stroop color‐word test—congruent and incongruent
Bruchle et al. 2021	Germany	23/18	33.3 ± 3.06	40.11 ± 3.6	GO/No‐Go Task—the reaction time and the correct response rate

**TABLE 2:** Table of intervention characteristics included in the study (*n* = 9).

Study	Intervention characteristics
Hoffman et al. 2008	Control group: drug group; experimental group: The exercise intervention was indoor treadmill aerobic exercise, conducted three times/week, more than 45 min, 16 weeks of high‐intensity exercise 70%–85% HRmax.
Imboden et al. 2020	Control group: stretching exercise; experimental group: The exercise intervention was indoor cycling aerobic exercise, three times a week for 6 weeks, 60%–75% HRmax moderate intensity exercise, the average duration of exercise was 45 min.
Khatri et al. 2001	Control group: drug group; experimental group: Treadmill brisk walking/jogging, three times/week, 16 weeks of high‐intensity exercise at 70%–85% HRmax, the exercise time was 45 min (10 min warm‐up exercise + 30 min treadmill brisk walking/jogging + 5 min cooling exercise).
Krogh et al. 2012	Control group: stretching exercise; the experimental group received a 12‐week high‐intensity exercise of 70%–80% HRmax, three times a week, and the exercise time was 45 min (10 min of warm‐up, 30 min of aerobic and 5 min of cooling).
Leleikiene et al. 2018	Control group: antidepressant drugs; experimental group: The exercise intervention was aerobic exercise, three times a week, for 4 weeks, 40%75% HRmax moderate intensity exercise, the exercise time was 40–50 min (10 min of warm‐up exercise + 25–30 min of aerobic endurance exercise and muscle strengthening exercise + 5–10 min of cooling exercise).
Leleikiene et al. 2018	Control group: antidepressant drugs; in the experimental group, the exercise intervention was resistance exercise, which was performed three times a week, for a period of four weeks of moderate intensity exercise at 40%–75% HRmax, and the exercise time was about 50 min (6–10 min veloergometer exercise + 25–30 min of three series of chest muscle and left and right leg strength training with 3 min interval between groups).
Olson et al. 2017	Control group: antidepressant or mood stabilizer; experimental group: the exercise intervention was treadmill or bicycle ergometer, conducted three times/week for 8 weeks at 40%–65% HRmax at moderate intensity, and the exercise time was about 45 min on the treadmill or bicycle.
I. Zhang et al. 2022	Control group: blank; experimental group: Tai Chi, two times a week for 12 weeks of low‐intensity exercise, the exercise time was about 90 min (3 min of jogging and 12 min of Tai chi step practice + 10 min of Tai chi kick + 40 min of 10 Tai chi + 15 min of Tai chi interaction + 10 min of Tai chi meditation).
Bruchle et al. 2021	Control group: blank; experimental group: coordination, endurance or strength training, three times a week for 3 weeks of low‐intensity exercise, exercise time of 60 min.

*Note*: For the convenience of table making, only the first author is registered, E/C represents experimental group/control group.

### 3.3 Quality Assessment of Included Literature

**FIGURE 2:**
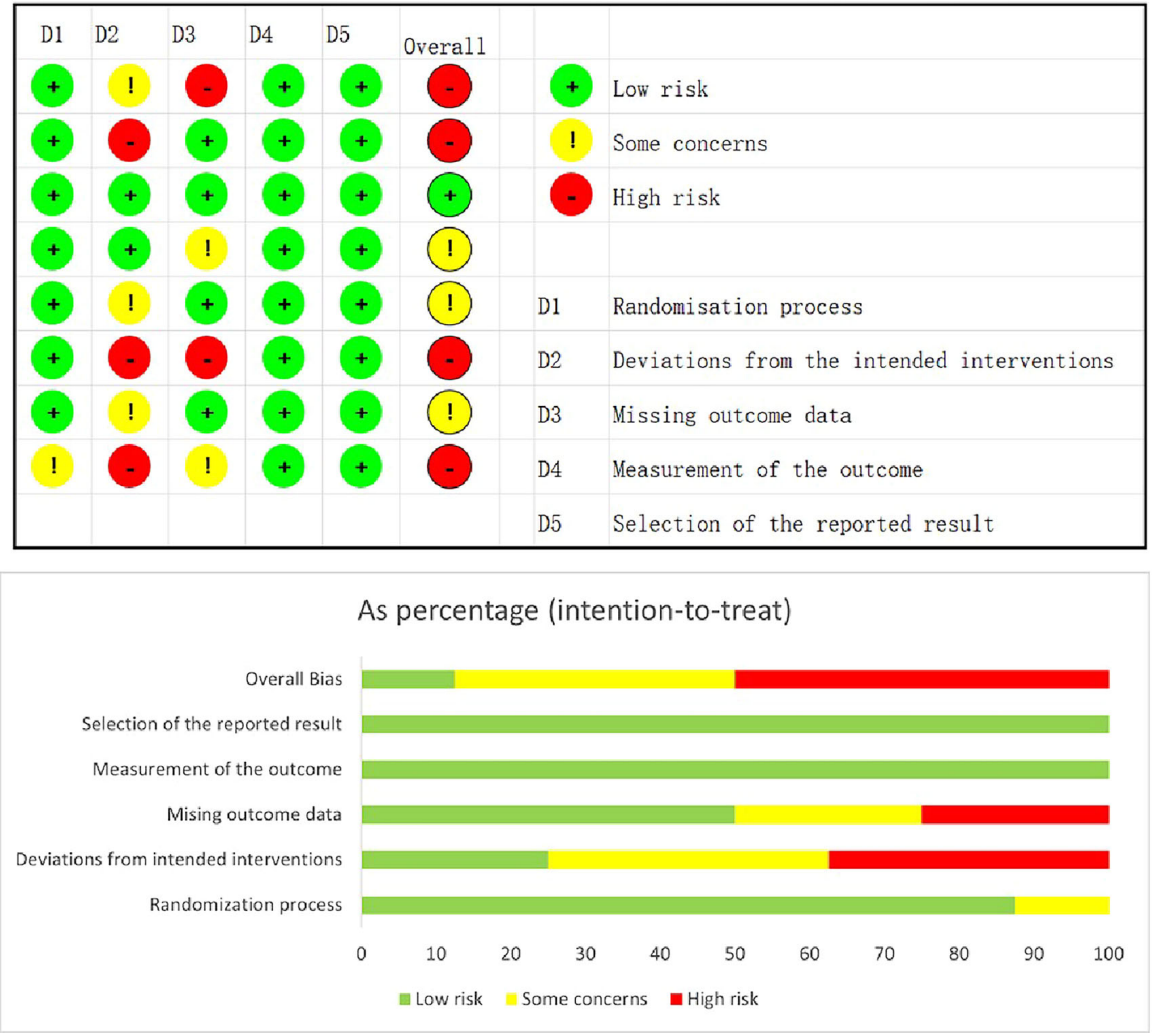
Methodological quality assessment included in the study (*n* = 8).

The risk of bias for each study was evaluated using the RoB 2.0 tool. Green means low risk of bias, yellow means moderate risk, and red means high risk. The total quality of each article is shown in the overall column. The results indicated that one study had a low risk of bias, three studies had a medium risk, and the remaining four studies had a high risk of bias, as depicted in Figure 2.

### 3.4 Meta‐Analysis Results

**FIGURE 3:**
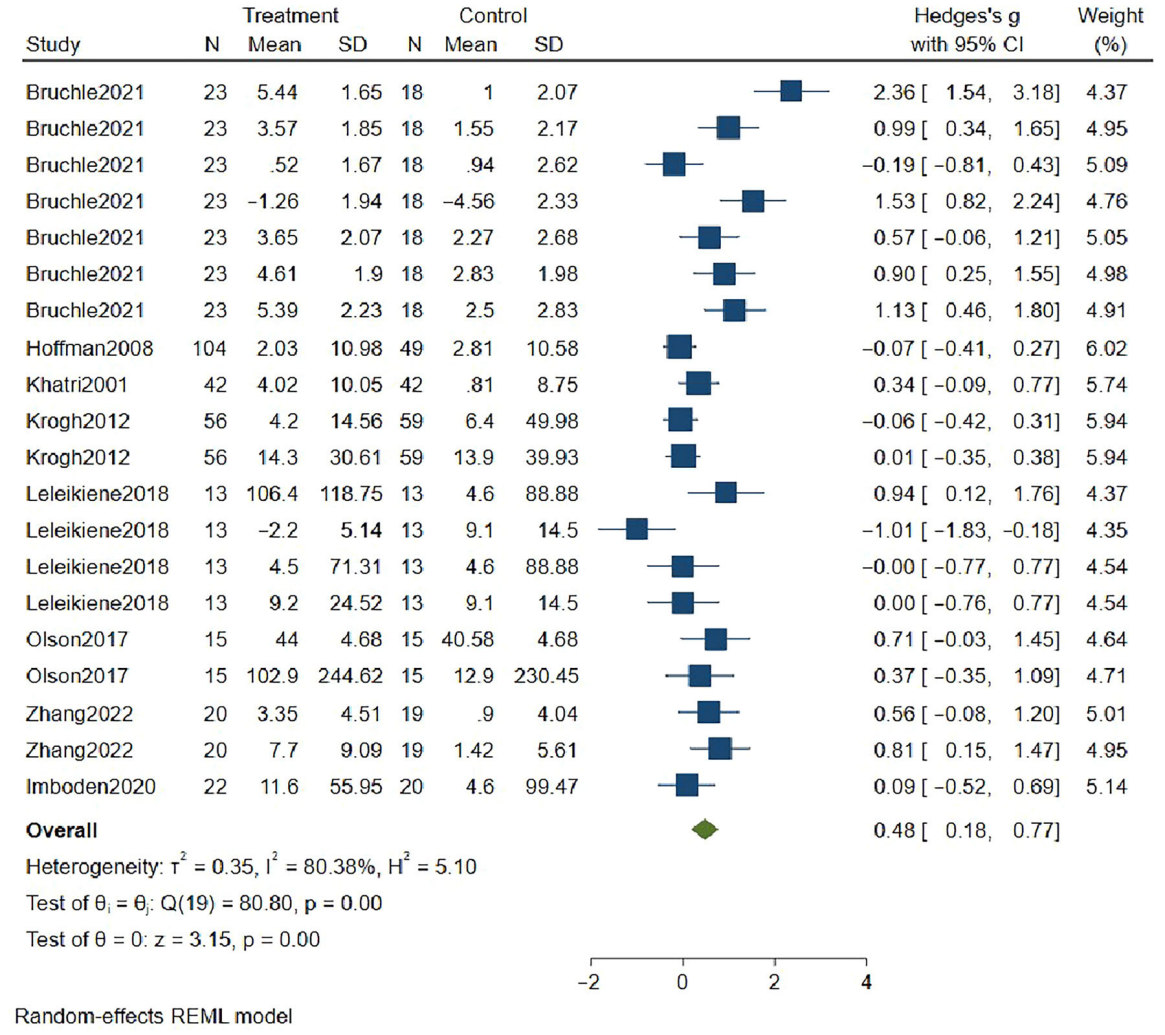
Forest diagram of the inhibitory function of exercise on patients with depression.

As shown in Figure 3, the nine included studies (Bruchle et al. 2021; Hoffman et al. 2008; Imboden et al. 2020; Khatri et al. 2001; Krogh et al. 2012; Olson et al. 2017; Leleikiene et al. 2018; I. Zhang et al. 2022) involving 1038 patients demonstrated effect sizes (Hedges's *g*) with a 95% confidence interval (CI). The size of the plotted squares reflects the statistical weight of each study. Blue squares represent individual studies, green diamonds represent summarized results, and the forest plots indicate that the results favored the experimental group. Meta‐analysis revealed significant heterogeneity (*I* ^2^ = 80.38%), and a random‐effects model was used to merge the data. A total of 20 effect sizes were included, resulting in a combined effect size of 0.48 (95% CI [0.18–0.77], *p* = 0.00), indicating that exercise significantly improved inhibitory function in depressed patients compared to the control group.

### 3.5 Sensitivity Analysis

**FIGURE 4:**
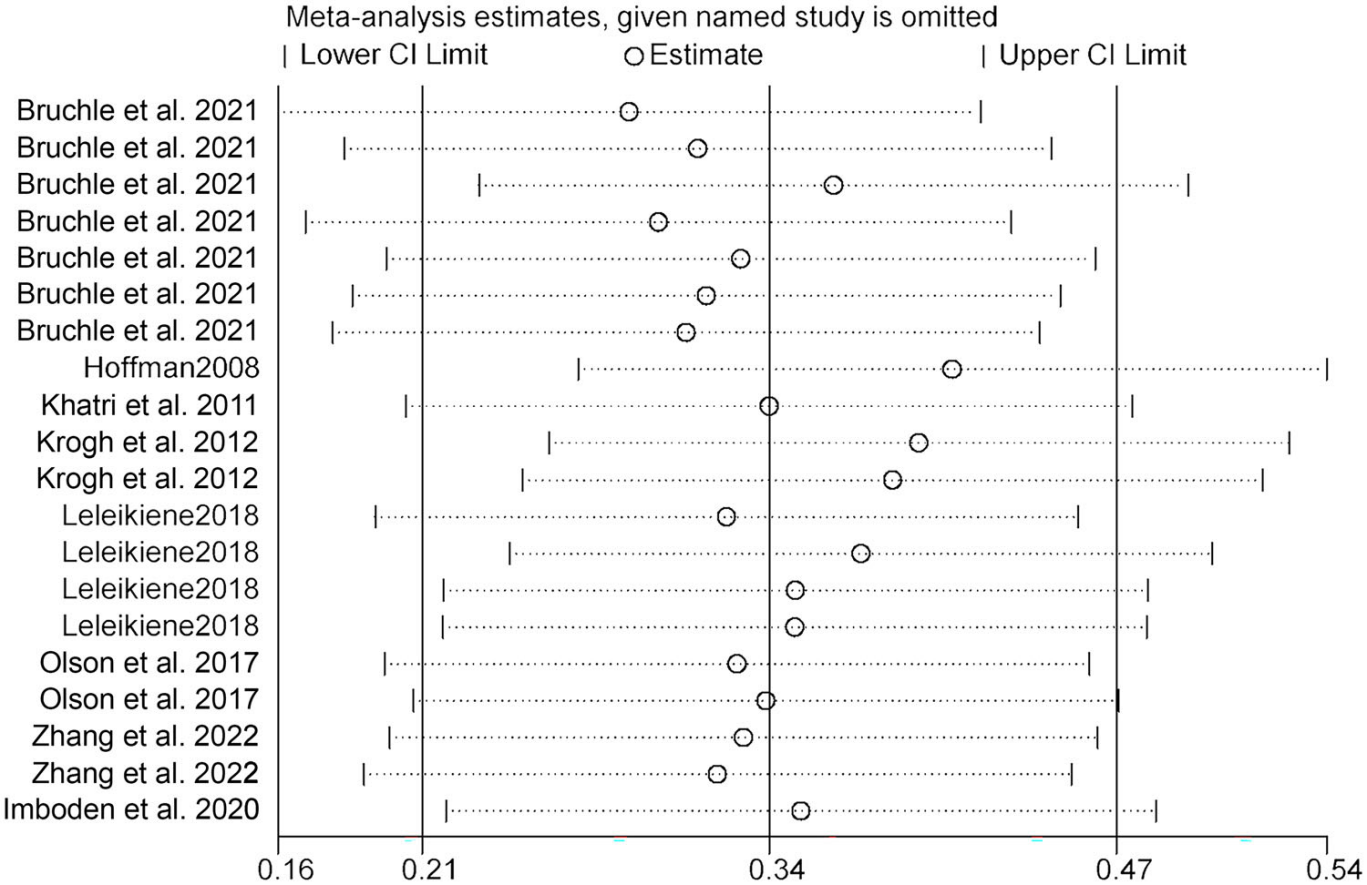
Sensitivity analysis of inhibitory function of exercise on patients with depression.

To determine whether the heterogeneity among studies was caused by a single study, a sensitivity analysis was performed by systematically excluding individual studies. The combined effect size of exercise on inhibitory function in patients with depression across all studies was SMD = 0.48 (95% CI: 0.18–0.77, P < 0.001, *I* ^2^ = 80.38%). After excluding individual studies, the range of the combined effect size SMD remained between 0.21 and 0.56, and the *I* ^2^ range remained between 79.64% and 83.33%, with *p* values consistently below 0.001. The results indicated that the data sensitivity of the study was relatively low, and the meta‐analysis results were stable and reliable. See Figure 4 for details.

### 3.6 Subgroup Analysis

To further explore the potential causes of heterogeneity, a subgroup analysis of potential moderator variables was conducted.

### 3.7 Measurement Tools

The subgroup analysis results show that, within the meta‐analysis, the inhibitory control measurement tools were categorized into three types. The Stroop task had the largest effect size, reaching a moderate effect size (SMD = 0.578), followed by the Flanker task (SMD = 0.535), and the Go/No‐Go task showed a small effect size (SMD = 0.348). This indicates statistically significant differences, suggesting that various testing tools may assess different components of inhibitory function differently.

#### 3.7.1 Mean Age

The mean age was categorized into two groups: >45 years and ≤45 years. It was found that there was statistical significance in the group with a mean age ≤45 years (SMD = 0.661, p = 0.002) (Hoffman et al. 2008; Khatri et al. 2001; Leleikiene et al. 2018; I. Zhang et al. 2022).

#### 3.7.2 Exercise Variables

Subgroup analyses based on exercise duration revealed that interventions lasting >45 min had a statistically significant effect (SMD = 0.936, *p* = 0.000) (Bruchle et al. 2021; I. Zhang et al. 2022), whereas interventions lasting ≤45 min did not show statistical significance (SMD = 0.087, *p* = 0.356) (Hoffman et al. 2008; Imboden et al. 2020; Khatri et al. 2001; Krogh et al. 2012; Olson et al. 2017; Leleikiene et al. 2018).

Subgroup analysis by exercise cycle found that intervention cycles ≤12 weeks had a statistically significant effect size (SMD = 0.525, *p* = 0.002) (Bruchle et al. 2021; Imboden et al. 2020; Khatri et al. 2001; Krogh et al. 2012; Olson et al. 2017; Leleikiene et al. 2018), while intervention cycles >12 weeks did not show statistical significance (SMD = 0.111, *p* = 0.586) (Hoffman et al. 2008; I. Zhang et al. 2022).

Subgroup analyses by exercise frequency revealed that interventions with a frequency of two times/week (SMD = 0.682, *p* = 0.004) (I. Zhang et al. 2022) and three times/week (SMD = 0.475, *p* = 0.007) (Bruchle et al. 2021; Hoffman et al. 2008; Imboden et al. 2020; Khatri et al. 2001; Krogh et al. 2012; Olson et al. 2017; Leleikiene et al. 2018) were statistically significant.

Subgroup analyses by inhibition function type found that interference inhibition (SMD = 0.563, p = 0.006) (Bruchle et al. 2021; Hoffman et al. 2008; Khatri et al. 2001; Krogh et al. 2012; Olson et al. 2017; I. Zhang et al. 2022) was statistically significant, whereas response inhibition (SMD = 0.348, p = 0.145) (Bruchle et al. 2021; Imboden et al. 2020; Leleikiene et al. 2018) was not.

Subgroup analysis by type of exercise found that AE (SMD = 0.027, *p* = 0.748) (Hoffman et al. 2008; Imboden et al. 2020; Khatri et al. 2001; Krogh et al. 2012; Olson et al. 2017; I. Zhang et al. 2022) did not have a statistically significant effect on inhibitory function. Other forms of exercise (resistance training, combined exercise) had a statistically significant effect (SMD = 0.863, *p* = 0.000) (Bruchle et al. 2021; Leleikiene et al. 2018).

Subgroup analyses by exercise intensity found that low‐intensity exercise had a statistically significant effect (SMD = 0.94, *p* = 0.000) (Bruchle et al. 2021; I. Zhang et al. 2022), whereas moderate to high‐intensity exercise did not show statistical significance (SMD = 0.09, *p* = 0.07) (Hoffman et al. 2008; Imboden et al. 2020; Khatri et al. 2001; Krogh et al. 2012; Olson et al. 2017; Leleikiene et al. 2018).

Subgroup analyses based on control group intervention modality found statistically significant effects for blank (SMD = 0.94, *p* = 0.00) (Bruchle et al. 2021; I. Zhang et al. 2022) and drug (SMD = 0.16, *p* = 0.01) (Hoffman et al. 2008; Khatri et al. 2001; Olson et al. 2017; Leleikiene et al. 2018) treatment groups. However, the stretching exercise control group did not show statistical significance (SMD = −0.01, *p* = 0.91) (Imboden et al. 2020; Krogh et al. 2012). Subgroup analyses based on intervention grouping revealed that the effect sizes for the blank control group (Table 3, Bruchle et al., 2021; Zhang et al., 2022) and pharmacological treatment group (Hoffman et al., 2008; Olson et al., 2017; Khatri et al. 2001; Leleikiene et al. 2018) were 0.94 and 0.16, respectively, both of which were statistically significant. However, the effect size for the stretching exercise group (Imboden et al., 2020; Krogh et al., 2012) was SMD = ‐0.01, P = 0.91, showing no statistical significance.

**TABLE 3:** Comparison table of classification of subgroup analysis of exercise on inhibitory function in patients with depression.

Research features		Adjusting variables		*I* ^2^
		ES, 95% CI	*p*	
Average age	≤45	0.661, 0.253, 1.070	0.002	82.57
	>45	0.206, −0.170, 0.583	0.283	67.30
Exercise duration	>45min	0.936, 0.494, 1.378	0.00	74.63
	≤45min	0.087, −0.098, 0.272	0.356	22.64
Period	>12week	0.111, −0.288, 0.509	0.586	53.16
	≤12week	0.525, 0.195, 0.855	0.002	79.73
Motion frequency	Two times per week	0.682, 0.223, 1.140	0.004	0
	Three times per week	0.457, 0.126, 0.788	0.007	82.76
Classification of inhibitory functions	Interference suppression	0.563, 0.163, 0.963	0.006	85.81
	Response inhibition	0.348, −0.120, 0.815	0.145	71.30
Motion type	Aerobic exercise	0.027, −0.136, 0.189	0.748	0.00
	Other sports	0.863, 0.492, 1.234	0.000	69.59
Exercise intensity	Low intensity	0.94, 0.49, 1.38	0.00	74.63
	Medium and above	0.09, −0.10, 0.27	0.07	22.64
Control group	Blank control	0.94, 0.49, 1.38	0.00	75.99
	Medical treatment	0.16, −0.20, 0.52	0.01	63.28
	Stretching exercise	−0.01, −0.24, 0.23	0.91	0

### 3.8 Publication Bias Test

**FIGURE 5:**
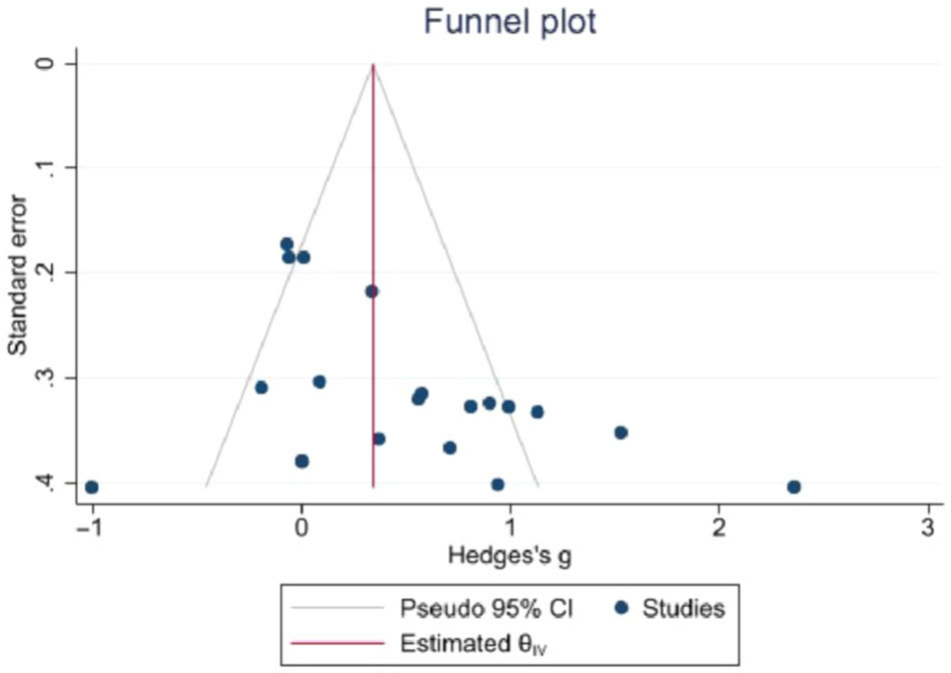
Funnel plot of inhibitory function of exercise on depression patients.

The funnel plot (Figure 5) of the effect of exercise on inhibitory function in patients with depression appears symmetrical. Egger's test result (*z* = 1.66, Prob > |*z*| = 0.0963) suggests that there is no publication bias in the studies.

### 3.9 Quality of Evidence Assessment

**FIGURE 6:**
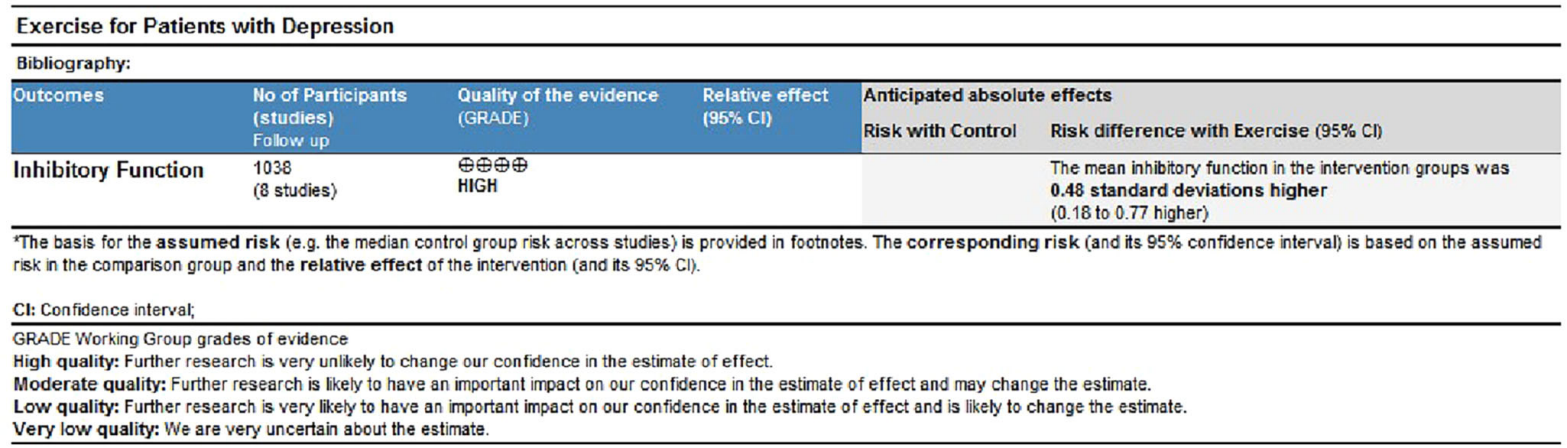
Quality of evidence on the improvement effect of exercise on inhibitory function in patients with depression.

As shown in Figure 6, the GRADEpro evidence grading system categorized the quality of evidence for the outcome indicators into four levels: high, intermediate, low, and very low. The evidence quality for the effect of exercise on improving inhibitory functioning in depressed patients was rated as high, suggesting that the actual effect is likely close to the estimate.

### 3.10 Adverse Events

No adverse events caused by exercise were reported in any of the eight included studies.

## 4 Discussion

The findings of this study indicate that exercise has a beneficial impact on inhibitory function in individuals with depression, with an effect size of SMD = 0.48. This is a small‐to‐medium effect size, as defined by the criteria proposed by Cohen. These results further substantiate the assertion that exercise has a significant positive effect on inhibitory function in individuals with depression. This finding is consistent with those of previous studies, which have confirmed that exercise has a profound effect on cognition and that it can accelerate the body's metabolism and improve cognitive function. Longitudinal studies (Bruchle et al. 2021) have also demonstrated the activation of the prefrontal lobe of the brain and the improvement status of inhibitory control function by exercise. Previous research has indicated that exercise may enhance activity in the prefrontal cortex of the brain, which is an important region for executive functions, including inhibitory functions (Basso and Suzuki 2017). An increase in prefrontal cortex activation results in enhanced inhibitory control. Second, it is hypothesized that exercise promotes neuroplasticity in the brain, including increased neuronal connectivity and improved synaptic function (Cotman, Berchtold, and Christie 2007). Such alterations may contribute to enhanced inhibitory function. With regard to the physiological and biochemical mechanisms that may be involved, it is possible that exercise affects the levels of neurotransmitters associated with mood and cognitive function. For example, increased serotonin and dopamine release have been observed (Meeusen and De Meirleir 1995). It is proposed that these neurotransmitter changes may contribute to improvements in inhibitory function and mood states. Additionally, previous research has indicated that exercise has the potential to reduce inflammatory processes within the body (Petersen and Pedersen 2005). Given the established link between inflammation and depression and cognitive decline, a combination of low‐intensity exercise may indirectly improve inhibitory function by reducing inflammation.

Eight studies were included, and one study showed a low risk because it provided a clear description of the randomization method used. Three studies showed a medium risk of bias because their randomization process did not elaborate on its details or the methodology addressed. Four other studies (Hoffman et al. 2008; Bruchle et al. 2021; Imboden et al. 2020; Olson et al. 2017) showed high risk of bias due to not allocating concealment in the randomization process. All literature was comparable with baseline comparisons, and all reported withdrawals and lost to follow‐up with moderate overall quality. The results were reliable. Also through the GRADE profiler software, we found that the quality of evidence for the effect of exercise on the improvement of inhibitory functioning in depressed patients was rated as advanced. Heterogeneity was found to be high in this study, and through subgroup and sensitivity analyses, it was found that the heterogeneity was derived from the inhibitory function measurement tool, type of exercise, duration of exercise, frequency of exercise, cycle of exercise, and intensity of exercise.

In this study, we found that the type of exercise was other exercise (RE or ME), the intervention duration was >45 min, the intervention period was <12 weeks, the frequency of intervention was two times/week, and the intensity of low‐intensity exercise was more effective on inhibitory functioning in patients with depression. Different types of exercise bring different benefits to individual inhibition. The type of exercise is an important variable of exercise. Most of the types of exercise in early studies (Jiang Wanting et al. 2022) belong to a single AE, and the interventions of the studies in recent years have been diversified; the intervention effects of RE and ME have also been confirmed. The results of the present study showed that AE, RE, and ME all had significant effects, but the effect size of AE was relatively small, which is consistent with the findings of Jun et al. (2014). The reason may be that there are more inhibitory control measurement tools in the included AE literature, and each measurement tool has different characteristics for inhibitory function, which may make the meta‐analysis effect size smaller compared to the other exercise types; exercise duration and exercise intensity are the specific embodiment of exercise loads in exercise practice, which are important factors affecting the effect of exercise. In this study, the duration was categorized into >45 min and ≤45 min, and the exercise intensity was categorized into low intensity and above‐medium intensity, and it was found that the intervention duration was statistically significant only for >45 min and that the exercise intensity of low‐intensity had a better effect on the intervention of inhibitory function in patients with depression, which is in line with the results of the studies conducted by Jones (2019), Knubben et al.’s (2007) study demonstrated significant improvements in both mood and cognitive functioning in depressed patients who exercised at low intensity. This may be due to the fact that low‐intensity endurance exercise causes changes in the concentration of some bioactive molecules such as pro‐adrenocorticotropic hormone, cortisol, catecholamines, opioid peptides, and cytokines (Knubben et al. 2007), which have been described as having a modulatory effect on mood. One study (Zhang et al., 2022) indicated that exercise with cognitive engagement (AE) provides additional improvements in self‐control, mental concentration, and emotional regulation compared to single AE, and these additional psychological adjustments may make cognitive‐engaged exercise more effective than conventional AE in alleviating depressive symptoms in the short term. The present study found that a frequency of two times/week was more effective in inhibiting function in depressed patients. This is consistent with studies by Chen (2016), Gapin, Labban, and Etnier (2011), and others (Han 2021). An intervention period of ≤12 weeks was more effective in inhibiting functioning in depressed patients. This is consistent with the studies of S. Zhang (2021) and Du (2021). Thompson (2020) found that participants in a long‐cycle exercise program were less effective than short‐ and medium‐cycle exercise in terms of mood improvement, particularly in terms of elevated inhibitory functioning. In a study comparing short‐ and medium‐cycle to long‐cycle exercise, researchers such as Garcia (2022) found that short‐ and medium‐cycle exercise was more effective in improving depressive symptoms and inhibitory function. This may be related to the adherence to short‐ and medium‐cycle exercise, making it easier for patients to stick with it. This could also be due to the specificity of the studies (Hoffman et al., 2008; Khatri et al., 2001), as participants in studies with durations longer than 12 weeks are primarily moderate depression patients. On the type of different control groups, it was found that there was no significant difference in the stretching exercise control group and significant difference in the blank control group and medicated exercise group compared to the experimental group. The possible reason for this is that the period, duration, and frequency of the intervention were the same for the stretching exercise and the experimental group, with different forms and intensities, and there were exercise benefits in both groups. Therefore, we suggest that future research should use both blank control and pharmacological interventions in the control group.

There are several limitations of this study: (1) This study included both Chinese and English literature at home and abroad and comprehensively searched the literature according to the classification of exercise forms in an effort to include more literature related to subject, but there is still a possibility that the relevant literature may have been missed and the number of literature is still limited. (2) This study was unable to control for the potential effects of confounding variables, which may have introduced a bias in the results. (3) The three literatures did not report the blinding situation in its entirety, and the difficulty of implementing the exercise intervention in a double‐blind fashion may have had an impact on the endpoint measurements. (4) Based on the clinical and demographic factors associated with adherence to an exercise program in patients with depression, patients were found to be closely related to duration of illness, symptom grade, sleep, diet, social support, and stress, but potential factors were not further analyzed due to incomplete demographic characterization of the included literature. (5) This study can only derive the dose relationship between resistance and combined exercise in patients with depression, and it is not possible to extend the dose relationship to other exercises.

## 5 Conclusion

This study found that exercise type of other exercise (RE or ME), intervention duration of >45 min, intervention period of ≤12 weeks, intervention frequency of two times/week, and intensity of low‐intensity exercise had better inhibitory functioning in depressed patients. The results have high reliability and provide more accurate exercise intervention programs for clinical practice and researchers. Meanwhile, more and more countries and regions have incorporated exercise into guidelines and policies for depression treatment, and in the future, governments and healthcare organizations should actively encourage depressed patients to improve their mental health by participating in physical activity. Future research and development will also further deepen the understanding of the role of exercise on the inhibitory function of depressed patients and explore more personalized, integrated, and technological exercise intervention programs. This will help to improve recovery outcomes, prevent depression, and provide better mental health support for people with depression.

## Author Contributions

**Zhihui Xu**: Conceptualization, methodology, validation, visualization, supervision, formal analysis, project administration, investigation, writing—review and editing, data curation, software, resources, funding acquisition, writing—original draft. **Cong Liu**: Conceptualization, investigation, writing—original draft, writing—review and editing, methodology, software. **Peng Wang**: Conceptualization, writing—review and editing, writing—original draft. **Xing Wang**: Conceptualization, data curation, supervision, resources, investigation, funding acquisition, writing—original draft. **Yuzhang Li**: Conceptualization, investigation, funding acquisition, writing—original draft, writing—review and editing, visualization, validation, methodology, project administration, formal analysis, software, resources, supervision, data curation.

## Conflicts of Interest

The authors declare no conflicts of interest

### Peer Review

The peer review history for this article is available at https://publons.com/publon/10.1002/brb3.70178.

## Data Availability

Research data are not shared.

## LITERATURE SEARCH PROCESS AND PICOS INCLUSION CRITERIA

### 1

**Table:**

	**Literature search process**
Data	Search process
PubMed, The Cochrane Libraryhe	#1“Exercise” [Mesh] OR “Aerobic exercise” [Title/Abstract] OR “physical exercise” [Title/Abstract] OR “resistance training” [Title/Abstract] OR “training” [Title/Abstract] OR “physical activity” [Title/Abstract] OR “sport” [Title/Abstract] OR “Ball sports” [Title/Abstract] OR “Physical and Mental Exercise” [Title/Abstract] OR “Martial Arts” [Title/Abstract] OR “yoga” [Title/Abstract] #2 “Depression” [Mesh] OR “Depressive disorder” [Title/Abstract] OR “Depressive symptom” [Title/Abstract] OR “Emotional depression” [Title/Abstract] OR “Depressive neurosis” [Title/Abstract] OR “Endogenous depression” [Title/Abstract] OR “Deurotic depression” [Title/Abstract] OR “Unipolar depression” [Title/Abstract] #3 “Inhibition function” [Mesh] OR “Cognition” [Title/Abstract] OR “Cognitive performance” [Title/Abstract] OR “Cognitive” [Title/Abstract] OR “executive function” [Title/Abstract] #4 Randomized controlled trial [Publication Type] OR “Randomized” [Title/Abstract] OR “controlled” [Title/Abstract] OR “Trial” [Title/Abstract] #5 #1 AND #2 AND #3 AND #4
Web of Science	#1 TS = (“Exercise” OR “Aerobic exercise” OR “physical exercise” OR “resistance training” OR “training” OR “physical activity” OR “sport” OR “Ball sports” OR “Physical and Mental Exercise” OR “Martial Arts” OR “yoga”) #2 TS = (“Depression” OR “Depressive disorder” OR “Depressive symptom” OR “Emotional depression” OR “Depressive neurosis” OR “Endogenous depression” OR “Deurotic depression” OR “Unipolar depression”) #3 TS = (“Inhibition function” OR “Cognition” OR “Cognitive performance” OR “Cognitive” OR “executive function”) #4 TS = (“Randomized controlled trial” OR “Randomized” OR “Controlled” OR “Trial”) #5 #1 AND #2 AND #3 AND #4
Embase	#1 “Exercise”[exp] OR “Aerobic exercise”[ab,ti] OR “physical exercise”[ab,ti] OR “resistance training” [ab,ti] OR “training” [ab,ti] OR “physical activity” [ab,ti] OR “sport” [ab,ti] OR “Ball sports” [ab,ti] OR “Physical and Mental Exercise” [ab,ti] OR “Martial Arts” [ab,ti] OR “yoga” [ab,ti] #2 “Depression”[exp] OR “Depressive disorder” [ab,ti] OR “Depressive symptom” [ab,ti] OR “Emotional depression” [ab,ti] OR “Depressive neurosis” [ab,ti] OR “Endogenous depression” [ab,ti] OR “Deurotic depression” [ab,ti] OR “Unipolar depression” [ab,ti] #3 “Inhibition function” [exp] OR “Cognition” [ab,ti] OR “Cognitive performance”[ab,ti]OR “Cognitive” [ab,ti] OR “executive function” [ab,ti] #4 “Randomized controlled trial” [exp] OR “Randomized” [ab,ti] OR“Controlled” [ab,ti] OR “Trial” [ab,ti] #5 #1 AND #2 AND #3 AND #4
CNKI	(Exercise + Aerobic exercise + resistance training + training + physical activity + sport + Anaerobic sports + Ball sports + Physical and Mental Exercise + Martial Arts + yoga)AND(Depression + Depressive disorder)AND(Inhibition function + Cognition + Cognitive performance + executive function + Cognitive)
Wan fang; CQVIP	(Exercise OR Aerobic exercise OR resistance training OR training OR physical activity OR sport OR Ball sports OR Physical and Mental Exercise OR Martial Arts OR yoga) AND (Depression OR Depressive disorder) AND (Inhibition function OR Cognition OR Cognitive performance OR executive function OR Cognitive)
	PICOS inclusion criteria
PICOS	Inclusion criteria
Participants	Subjects conforming to any diagnostic standard as per the International Classification of Diseases (ICD) or the Diagnostic and Statistical Manual of Mental Disorders (DSM).
Intervention	The intervention for the experimental group consists of exercise or exercise‐based intervention in addition to what the control group receives.
Control	The control group receives conventional treatment (pharmacotherapy, stretching exercises, or no intervention).
Outcome	Indicators related to inhibitory function, including: 1. Stroop test; 2. Go/No‐Go test; 3. Flanker test.
Study design	Randomized controlled trial

Abbreviations: C:comparison;I:intervention; O:outcome;P:population; S:Study design.
